# Gene Signatures of Early Response to Anti-TNF Drugs in Pediatric Inflammatory Bowel Disease

**DOI:** 10.3390/ijms21093364

**Published:** 2020-05-09

**Authors:** Sara Salvador-Martín, Irene Raposo-Gutiérrez, Víctor Manuel Navas-López, Carmen Gallego-Fernández, Ana Moreno-Álvarez, Alfonso Solar-Boga, Rosana Muñoz-Codoceo, Lorena Magallares, Eva Martínez-Ojinaga, María J. Fobelo, Antonio Millán-Jiménez, Alejandro Rodriguez-Martinez, Concepción A. Vayo, Cesar Sánchez, Mar Tolin, Ferrán Bossacoma, Gemma Pujol-Muncunill, Rafael González de Caldas, Inés Loverdos, José A. Blanca-García, Oscar Segarra, Francisco J. Eizaguirre, Ruth García-Romero, Vicente Merino-Bohórquez, María Sanjurjo-Sáez, Luis A. López-Fernández

**Affiliations:** 1 Pharmacy Department, Hospital General Universitario Gregorio Marañón, Instituto de Investigación Sanitaria Gregorio Marañón, 28007 Madrid, Spain; sara.salvador@iisgm.com (S.S.-M.); Irene.raposo.gutierrez@gmail.com (I.R.-G.); maria.sanjurjo@salud.madrid.org (M.S.-S.); 2 Pediatric Gastroenterology and Nutrition Unit, Hospital Regional Universitario de Málaga, IBIMA Multidisciplinary Group for Pediatric Research, 29010 Málaga, Spain; victorm.navas.sspa@juntadeandalucia.es; 3 Pharmacy Department, Hospital Regional Universitario de Málaga, 29010 Málaga, Spain; Cgallegofernandez@gmail.com; 4 Pediatric Gastroenterology Unit, Department of Pediatrics, A Coruña University Hospital, 15006 A Coruña, Spain; ana.moreno.alvarez@sergas.es (A.M.-Á.); alfonso.solar.boga@sergas.es (A.S.-B.); 5 Department of Pediatric Gastroenterology, Hospital Infantil Universitario Niño Jesús, 28009 Madrid, Spain; rosana.munoz@salud.madrid.org; 6 Department of Pediatric Gastroenterology, University Hospital La Paz, 28046 Madrid, Spain; lorena.magallares.garcia@gmail.com (L.M.); ojinaganodal@gmail.com (E.M.-O.); 7 Pharmacy Service, Hospital Virgen de Valme, 41014 Sevilla, Spain; mariaj.fobelo.sspa@juntadeandalucia.es; 8 Pediatric Gastroenterology Unit, Hospital Virgen de Valme, 41014 Sevilla, Spain; millan472@gmail.com; 9 Pediatric Gastroenterology, Hepatology and Nutrition Unit, Hospital Universitario Virgen del Rocio, 41013 Seville, Spain; alejandro.rodriguez.m.sspa@juntadeandalucia.es; 10 Pharmacy Service, Hospital Universitario Virgen del Rocio, 41013 Seville, Spain; concepcion.alvarezvayo.sspa@juntadeandalucia.es; 11 Gastroenterology Unit, Hospital General Universitario Gregorio Marañón, Instituto de Investigación Sanitaria Gregorio Marañón, 28007 Madrid, Spain; sangoros@hotmail.com (C.S.); marth81@gmail.com (M.T.); 12 Fundació Sant Joan de Déu, Fundació Salut Emporda, 08950 Barcelona, Spain; fbossacoma@sjdhospitalbarcelona.org; 13 Department of Pediatric Gastroenterology, Hepatology and Nutrition, Hospital Sant Joan de Déu, 08950 Barcelona, Spain; gpujol@sjdhospitalbarcelona.org; 14 Pediatric Gastroenterology Unit, Hospital Reina Sofía, 14004 Córdoba, Spain; rgonzalezdecaldasmarchal@gmail.com; 15 Pediatric Gastroenterology, Hepatology and Nutrition Unit, Hospital de Sabadell, Corporació Sanitària Universitària Parc Taulí, 08208 Barcelona, Spain; ines.loverdos@gmail.com; 16 Pediatric Gastroenterology Unit, Hospital Puerta del Mar, 11009 Cadiz, Spain; jablanca@hotmail.com; 17 Pediatric Gastroenterology Unit, Hospital Universitario Vall d’Hebrón, 08035 Barcelona, Spain; osegarra@vhebron.net; 18 Pediatric Gastroenterology Unit, Hospital Universitario Donostia, 20014 San Sebastián, Spain; franciscojavier.eizaguirrearocena@osakidetza.eus; 19 Pediatric Gastroenterology Unit, Hospital Infantil Miguel Servet, 50009 Zaragoza, Spain; Ruthgarciaromero@yahoo.es; 20 UGC Pharmacy Department, Hospital Virgen de la Macarena, 41009 Sevilla, Spain; vicente.merino.sspa@juntadeandalucia.es

**Keywords:** biomarker, gene expression, infliximab, adalimumab, ulcerative colitis, Crohn’s disease

## Abstract

Around a 20–30% of inflammatory bowel disease (IBD) patients are diagnosed before they are 18 years old. Anti-TNF drugs can induce and maintain remission in IBD, however, up to 30% of patients do not respond. The aim of the work was to identify markers that would predict an early response to anti-TNF drugs in pediatric patients with IBD. The study population included 43 patients aged <18 years with IBD who started treatment with infliximab or adalimumab. Patients were classified into primary responders (*n* = 27) and non-responders to anti-TNF therapy (*n* = 6). Response to treatment could not be analyzed in 10 patients. Response was defined as a decrease in over 15 points in the disease activity indexes from week 0 to week 10 of infliximab treatment or from week 0 to week 26 of adalimumab treatment. The expression profiles of nine genes in total RNA isolated from the whole-blood of pediatric IBD patients taken before biologic administration and after 2 weeks were analyzed using qPCR and the 2^−∆∆Ct^ method. Before initiation and after 2 weeks of treatment the expression of *SMAD7* was decreased in patients who were considered as non-responders (*p* value < 0.05). Changes in expression were also observed for *TLR2* at T0 and T2, although that did not reach the level of statistical significance. In addition, the expression of *DEFA5* decreased 1.75-fold during the first 2 weeks of anti-TNF treatment in responders, whereas no changes were observed in non-responders. Expression of the *SMAD7* gene is a pharmacogenomic biomarker of early response to anti-TNF agents in pediatric IBD. *TLR2* and *DEFA5* need to be validated in larger studies.

## 1. Introduction

Inflammatory bowel disease (IBD), which includes ulcerative colitis (UC) and Crohn’s disease (CD), is an autoimmune disorder whose pathogenesis genetics and environmental factors play a key role [1,2]. Around 20–30% of IBD patients are diagnosed before they are 18 years old. Pediatric IBD (pIBD) is associated with more extensive disease and a more complicated course than adult-onset IBD [3]. These characteristics and the fact that children have to live longer with the existing therapy underline the need for optimization of treatments of pIBD.

Biological therapy, mainly anti-TNF agents, has revolutionized the treatment of IBD and other inflammatory diseases. The phenotype of pIBD usually leads to earlier use of biological therapy in children [4]. However, and in spite of the usefulness of anti-TNF agents, failure of treatment is a major area for improvement. Thus, after 10 weeks of treatment with infliximab in children with moderate-to-severe CD, 11.6% did not respond and 41.1% did not achieve clinical remission [5]. Failure rates were even higher at week 54, with no response in 46.5% and a failure to have a clinical response in 44.2%. However, other biological drugs approved in adults, such as vedolizumab (anti-integrin α4β7) or ustekimumab (an anti-IL-23), are used off-label in children when treatment with infliximab and adalimumab fails [6].

Mucosal healing is the best outcome measure in pIBD and it is used to compare the efficacy of different biological therapies [7]. However, given that pIBD is a chronic disease, frequent biopsies would be required to monitor response and because this is unfeasible, the use of non-invasive biomarkers is highly recommended.

Serological or fecal biomarkers, such as trough serum anti-TNF levels and antidrug antibodies and fecal calprotectin, have been related to the response to anti-TNF treatment and are usually monitored in clinical practice [8,9,10,11]. However, their predictive value during the first 2 weeks of anti-TNF treatment is limited and more biomarkers are needed. Trough serum anti-TNF levels 6 weeks after initiation of treatment were recently reported to predict remission [12]. Several DNA polymorphisms have been associated with a response to anti-TNF drugs. However, most of these associations are weak and not valid for use in clinical practice [13,14,15,16,17,18]. In addition, most of them have been identified in adults but have not been tested in children yet.

The identification of genomic biomarkers could help us to identify groups of children who are less likely to respond to anti-TNFs before the beginning of treatment. The expression profile of several genes in inflamed tissues have been associated with a response to anti-TNF agents in adults diagnosed with IBD or other autoimmune disorders [19,20,21,22,23]. However, little is known about this subject in children with IBD. A recent study analyzing blood transcriptome biomarkers identified genes that were differentially expressed in children with clinically active IBD (but not adults) and healthy donors [24]. Unfortunately, most of these studies used biopsied inflamed tissue as their sample, and, as biopsy is an invasive technique, the biomarkers obtained could not be monitored. Gene expression biomarkers from whole blood could prove advantageous, although only a few studies have used this approach, and, again, the study population only comprised adults [25,26].

In the present study, we measured the expression of the genes *SMAD7*, *TNF*, *TLR2*, *TNFRSF1B*, *TBX21*, *DEFA5*, *IL11*, *TREM1*, and *OSM* (previously studied as related to the anti-TNF response or IBD in adults) [25,27,28,29,30,31] in the whole blood of children with IBD before and after 2 weeks of anti-TNF treatment. The aim was to evaluate whether these genes could be very early pharmacogenomics biomarkers of response to anti-TNF agents in pIBD.

## 2. Results

### 2.1. Patients Characteristics

**Figure 1:**
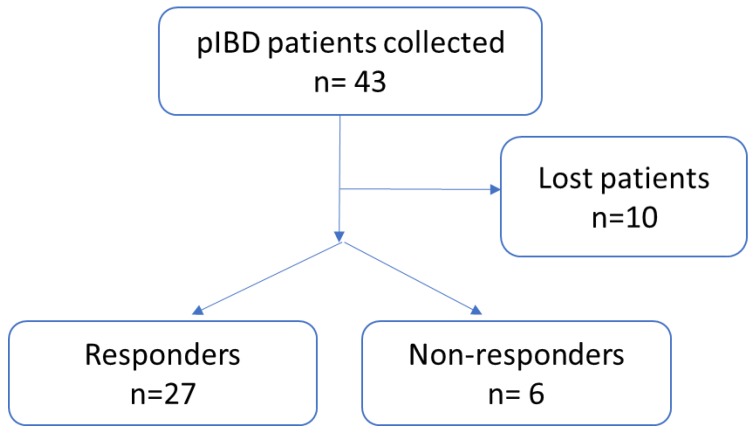
Flow chart showing selection of study patients. pIBD, pediatric inflammatory bowel disease. Some patients were lost because paxgene could not be collected at time 0 or 2, some data were missing or the quality of cDNA was insufficient.

Forty-three patients met the inclusion criteria and were included in the study (Figure 1). Twenty-seven responded to anti-TNF treatment according to the established criteria, six did not respond and ten were excluded from further analysis due to missing samples, missing clinical data needed to evaluate the response, or due to low RNA/cDNA quality.

The anti-TNF treatment failure rate was 18.2%, which was much lower than expected. The characteristics of both groups of patients are summarized in Table 1.

Eighteen patients received infliximab and fifteen patients were treated with adalimumab. The only statistically significant characteristic between the two response groups was the PCDAI at the start of treatment (PCDAI 30 in responders vs. 15 in non-responders, *p* value = 0.013) (Table 1).

### 2.2. Differential Gene Expression in the Response of Anti-TNF Agents Prior to Starting Treatment

In order to identify differential gene expression profiles that could be used as markers of response to anti-TNF treatment, we compared the relative expression of *TLR2*, *TNF*, *TNFRSF1B*, *IL11*, *TBX21*, *SMAD7*, *TREM1*, *OSM* and *DEFA5* in responders and non-responders immediately prior to the first administration of the anti-TNF agents. The analysis was performed using responders (R-T0) as the biological reference group. The *SMAD7* mRNA expression was decreased in non-responders compared to responders (–2.1-fold. *p* value < 0.05). In addition, a decrease in expression of *DEFA5* (–2.0-fold) and *TLR2* (–1.8-fold) were observed in non-responders compared with responders (Supplemental Table S1), but these changes were not statistically significant.

**Figure 2:**
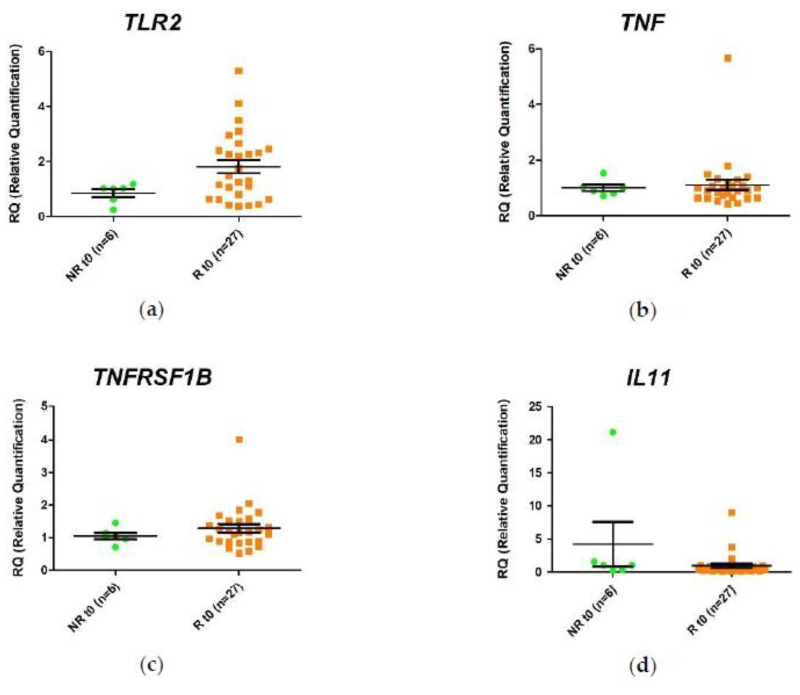

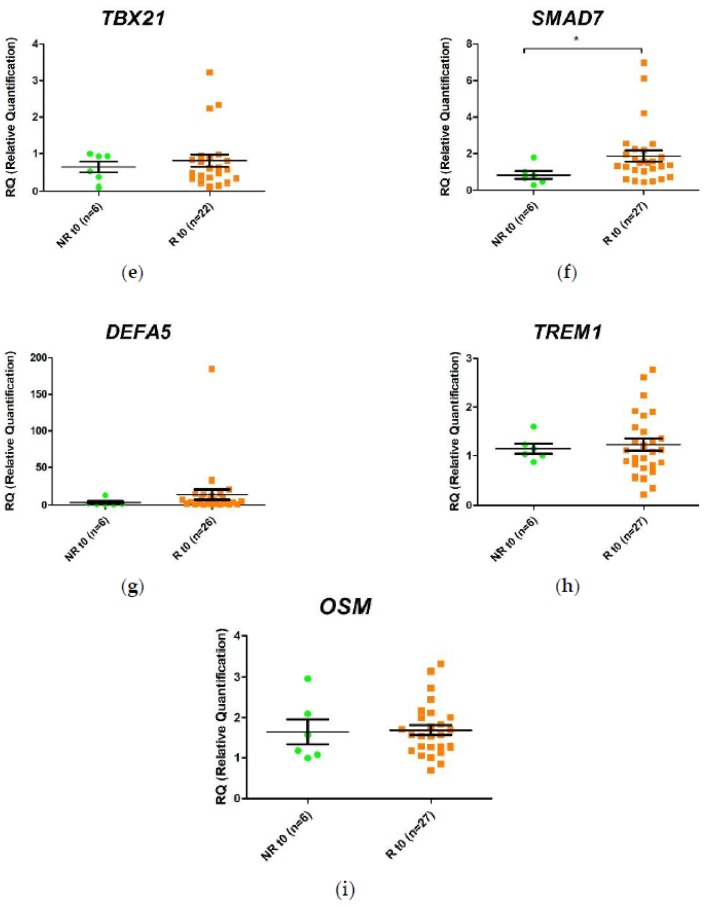
Relative expression levels of the (**a**) *TLR2*, (**b**) *TNF*, (**c**) *TNFRSF1B*, (**d**) *IL11*, (**e**) *TBX21*, (**f**) *SMAD7*, (**g**) *DEFA5*, (**h**) *TREM1*, and (**i**) *OSM* genes in responders (R) and non-responders (NR) at time 0. Expression values were normalized to *HPRT1* and *GAPDH* genes. Values are expressed as mean (horizontal line), standard error of the mean (SEM). *n*, sample size. * *p* value < 0.05 vs. control (unpaired *t*-test).

These results and the inter-sample variability are observed more clearly when the relative expression values of each sample are represented graphically (Figure 2).

### 2.3. Differential Gene Expression in Response to Anti-TNF Agents at Week 2 Post-Treatment

Gene expression was analyzed at week 2 post-treatment in responders vs. non-responders, as in the previous case, using the responders as the biological reference group (R-T2). The results showed a lower expression of *SMAD7* (–1.8-fold, *p* value < 0.05) in non-responders compared with responders (Supplemental Table S2).

**Figure 3:**
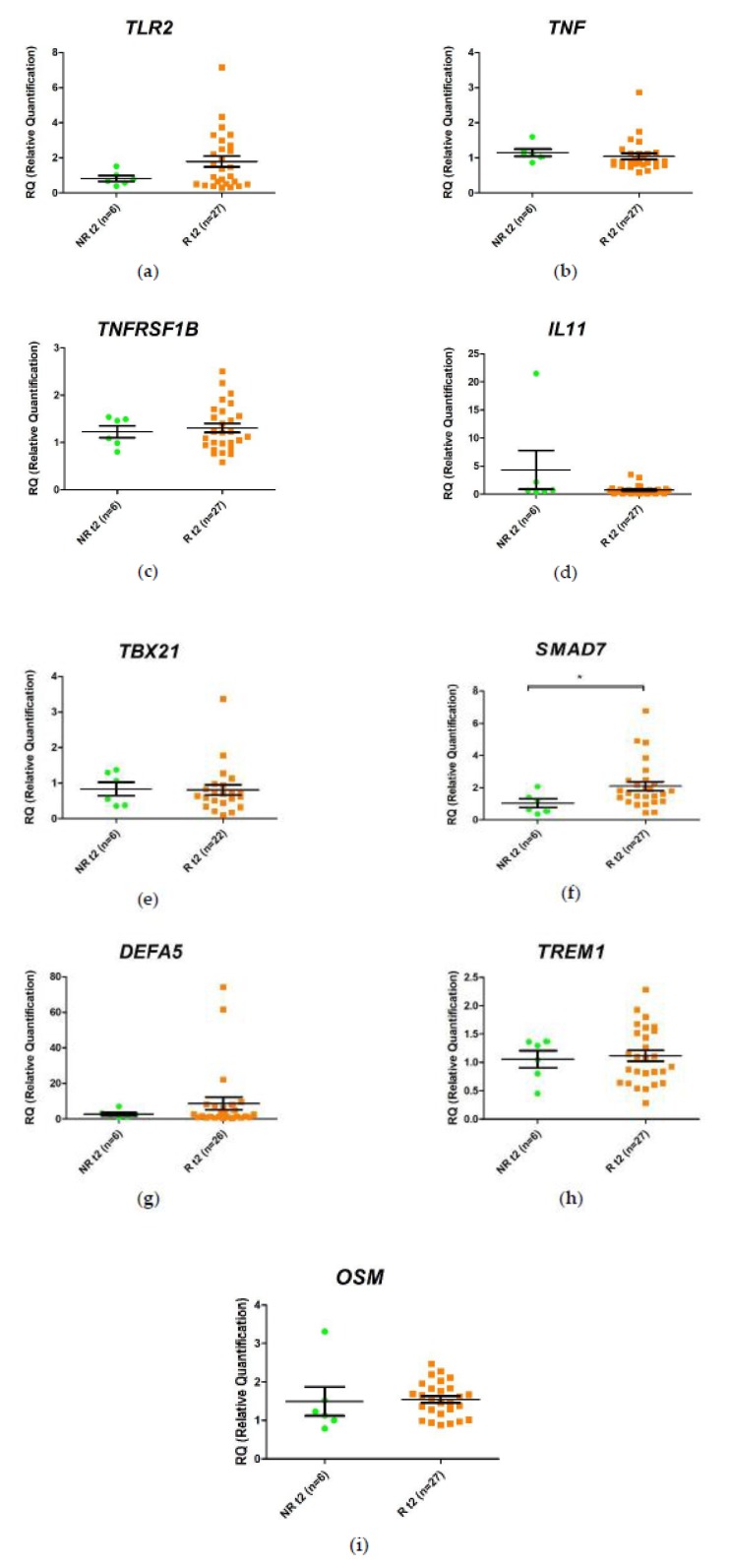
Relative expression levels of the (**a**) *TLR2*, (**b**) *TNF*, (**c**) *TNFRSF1B*, (**d**) *IL11*, (**e**) *TBX21*, (**f**) *SMAD7*, (**g**) *DEFA5*, (**h**) *TREM1*, and (**i**) *OSM* genes in responders (R) and non-responders (NR) at 2 weeks after anti-TNF administration. Expression values were normalized to *HPRT1* and *GAPDH* genes. Values are expressed as mean (horizontal line), standard error of the mean (SEM). *n*, sample size. * *p* value < 0.05 vs. control (unpaired *t*-test).

These results and inter-sample variability were observed more clearly when the relative expression values of each sample were represented graphically (Figure 3). The expression of *TLR2* was lower (–1.6-fold) in non-responders, although this difference was not statistically significant.

### 2.4. Differential Change in Gene Expression during the First 2 Weeks of Anti-TNF Treatment

**Figure 4:**
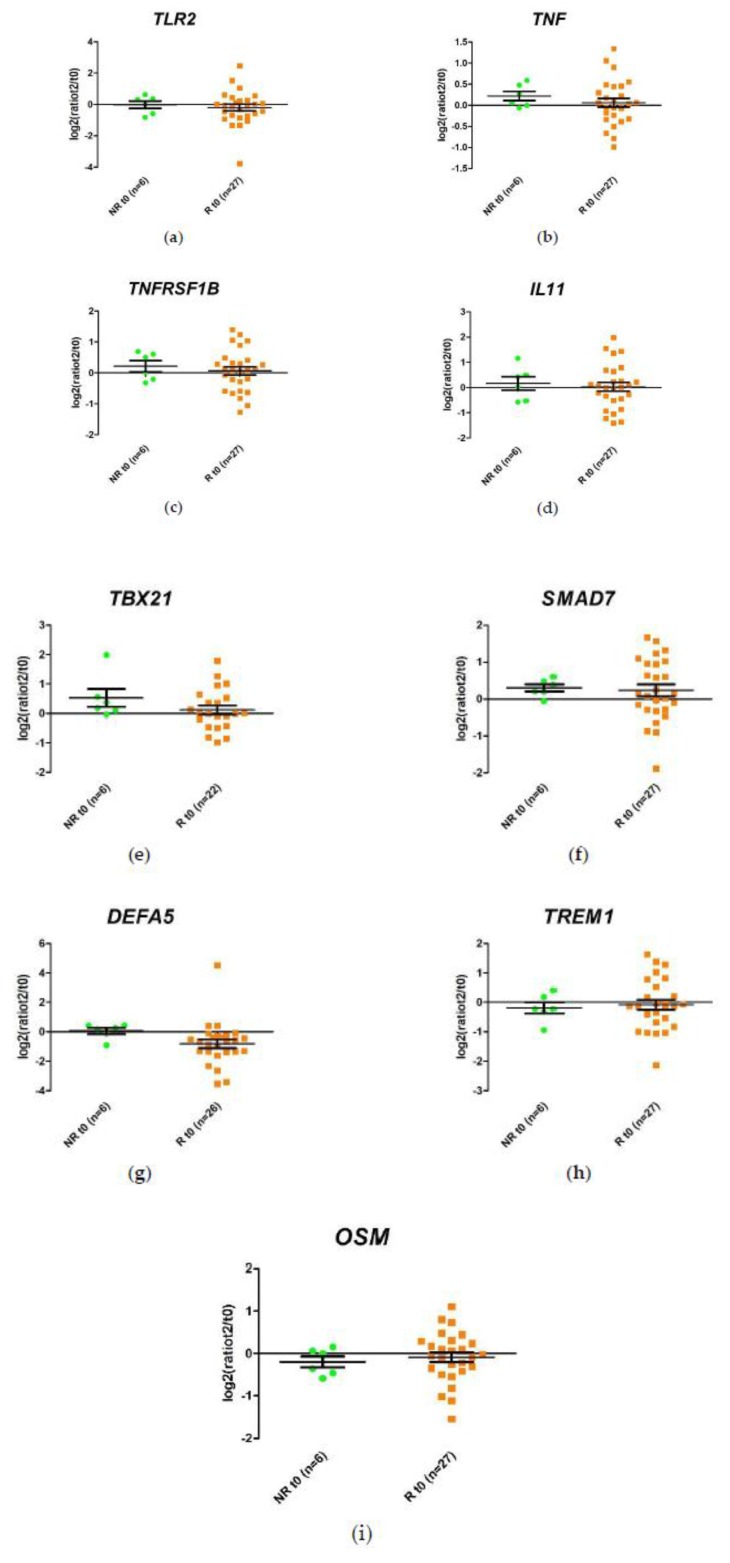
Schematic representation of the ratio between relative expression levels at week 2 (t2) and week 0 (t0) (t2/t0) of the (**a**) *TLR2*, (**b**) *TNF*, (**c**) *TNFRSF1B*, (**d**) *IL11*, (**e**) *TBX21*, (**f**) *SMAD7*, (**g**) *DEFA5*, (**h**) *TREM1, and* (**i**) *OSM* genes in responders (R) and non-responders (NR). Expression values were normalized to *HPRT1* and *GAPDH* genes. Values are expressed as mean (horizontal line), standard error of the mean (SEM). *n*, sample size. * *p* value < 0.05 vs. control (unpaired *t*-test).

We studied the changes in gene expression produced during the first 2 weeks of anti-TNF treatment, which differed between responders and non-responders. This difference was measured as the ratio of the expression levels for each patient at time 2 weeks (t2) and before initiation of treatment (t0) (t2/t0). The expression of *DEFA5* decreased 1.75-fold after the first 2 weeks of anti-TNF treatment in responders, whereas no changes were observed in non-responders, although this difference was not statistically significant. These results and inter-sample variability were observed more clearly when the relative expression values of each sample were represented graphically (Figure 4).

### 2.5. Prediction of Response to Anti-TNF Therapy Based on SMAD7 Expression Prior to Starting Treatment

The *SMAD7* mRNA expression at t0 was decreased in non-responders compared to responders (Figure 1). The positive predictive value (PPV) for response to anti-TNF treatment was 45%, the negative predictive value (NPV) was 95%. Sensitivity was 83% and specificity 77% using a cut-off of 1 for *SMAD7* relative expression in whole blood prior to treatment. The diagnostic odds ratio, which measures the effectiveness of a test independently of the prevalence, was 16.14. In addition, with a prevalence of 0.18, the positive likelihood ratio (+LR) was 3.75 (95% CI 1.70–8.27), and the negative LR (–LR) was 0.21 (95% CI 0.03–1.32).

## 3. Discussion

Although the anti-TNF agents infliximab and adalimumab have revolutionized the treatment of autoimmune diseases, a significant percentage of patients do not respond to this therapy [8]. Knowing in which patient therapy is more likely to fail after 2 weeks or even before initiation would allow therapy to be personalized, thus making it safer and more efficient, especially in children, who are necessarily treated for longer. There is currently, no other approved biological treatment in Europe for children with inflammatory bowel disease (infliximab and adalimumab for CD and infliximab for UC) which makes optimization of treatment especially important in this population. In addition, the genetics of pIBD differs from adult IBD highlighting the relevance for finding specific biomarkers for children. Thus, polymorphisms in genes, such as *NOD2/CAR15*, *ATG16L1*, *IL23R*, and *IL10R* have been involved in susceptibility to pIBD or to very-early-onset ulcerative colitis [32,33]. Differences in serum levels of clusterin and ceruloplasmin between children and adults with IBD have also been found [34]. Concerning differences in response to treatment, SNPs in *ATG16L1*, *CDKAL1*, *ICOSLG*, *BRWD1*, and *HLA-DQA1* have been associated with an anti-TNF response specifically in children [35]. However, concerning gene expression, specific profiles have been associated with a response to anti-TNF agents in adults with IBD, but not in children [19,20,23]. To our knowledge, the present study is the first to analyze gene expression profiles in children with IBD based on biomarkers of response to anti-TNF agents. We identified *TNF*, *SMAD7*, and *DEFA5* to be potential pharmacogenomic markers for early response to anti-TNF drugs in pediatric patients with IBD. This discovery is a first step for what in the future may constitute a minimally invasive routine test in daily clinical practice that could help to select the best treatment options for these patients. Several authors have included these three genes in their gene expression studies in the search for a biomarker that would enable us to predict the response to anti-TNF agents in patients with various diseases, mainly rheumatoid arthritis [21,22,26,36,37]. None of these three genes proved to be a biomarker of response. However, none of the aforementioned studies were performed in adults, thus suggesting that there are differences in expression that are specific to children.

Our results showed that expression of *SMAD7* mRNA was downregulated in the peripheral blood cells of non-responders after 2 weeks of anti-TNF treatment. SMAD7 negatively regulates phosphorylation of the SMAD2/SMAD3 complex, which is necessary for TGF-ß signaling [38,39]. Several studies have linked deregulation of the TGF-β/SMAD pathway with the pathogenesis of many autoimmune diseases, including psoriasis [40] and IBD. In the case of IBD, the inflammation associated with CD is characterized by a decrease in the immunosuppressive activity of TGF-β as a result of overexpression of *SMAD7* in the affected regions of the intestine [41]. However, other authors suggest that inhibition of *SMAD7* increases values for proinflammatory cytokines, such as IL-2, thus driving inflammation and enhancing the proinflammatory Th17 immune response [42]. Expression of *SMAD7* also decreases in the peripheral blood mononuclear cells of patients with multiple sclerosis [43,44]. Recently, our group confirmed this observation and extended its findings to adults diagnosed with CD [45]. We suggest that highly-expressed *SMAD7* cells are enriched at inflammation sites and, in parallel, poorly expressed *SMAD7* cells are enriched in peripheral blood. TGF-β is a cytokine with important immunomodulatory properties that control the activation status of all immune system cells and contributes to peripheral differentiation of regulatory cells (anti-inflammatory cells) and Th17 T cells (pro-inflammatory cells) [46]. This dual role of TGF-ß makes it difficult to understand the exact molecular mechanisms behind its regulation. In any case, expression of *SMAD7* is a valuable biomarker of early response to anti-TNF response in children with IBD, although this should be validated in a larger cohort.

In addition, our results showed that *DEFA5* mRNA levels tend to decrease between week 0 and 2 in responders, but not in non-responders. DEFA5 is a cytotoxic peptide that is produced in large quantities by Paneth cells. It is involved in the control of intestinal microbiota and plays a crucial role in its homeostasis [47]. DEFA5 and DEFA6 are reduced in the small bowel of patients with CD [47]. Recently, Hu et al. [48] demonstrated the implication of activating transcription factor 4 (ATF4) in IBD. ATF4 is decreased in the inflamed intestinal mucosa of patients with IBD, and its deficiency in mice leads to reduced glutamine absorption and decreased expression of antimicrobial peptides such as DEFA5 in the Paneth cells of the ileum. For this reason, it is difficult to determine the cause of the decrease in *DEFA5* mRNA expression in blood during the first 2 weeks of anti-TNF treatment in pediatric responders. As for *SMAD7*, expression in inflamed tissues and in blood may be increased in one and decreased in the other.

*TREM1* and *TNF* genes have been associated with a response to anti-TNF drugs in adults [25,49]. Furthermore, TNF level in stimulated PBMCs has been described as a good marker of response to infliximab in adults with IBD [50]. In our study, no differences were observed for these genes in children treated with infliximab or adalimumab. This lack of correlation could be due to low sample size or other study limitations. However, inherent differences in gene expression between children and adults in the response to anti-TNF drugs could also be the cause.

The main limitation of the study is its sample size, which is insufficiently large to take account of the wide interindividual variability in gene expression observed in blood [22,26]. Gene expression may also vary with respect to the two drugs used, adalimumab and infliximab. This low sample size also prevents us from knowing whether the difference observed in the PCDAI index between responders and non-responders is or may be a direct cause of the response. Sample size also made it impossible to analyze differences between CD and UC and precluded correction for multiple testing Additionally, an effect of drug exposure on outcomes cannot be ruled out, although its impact was minimized due to monitoring of trough serum anti-TNF levels during follow-up in most of these patients. However, the analysis performed in this paper was preliminary.

In addition, comparison with similar studies is difficult for two reasons. First, there are no data on children. Second, response criteria differ with studies. Thus, for example, the response may be based on various parameters, such as endoscopic and histological healing [19], or the decrease in the Mayo score [23].

More studies involving larger populations are necessary to confirm these findings. Nevertheless, the various blood biomarkers of response to anti-TNF agents identified to date could help to improve the therapy in pIBD.

In summary, we verified that low *SMAD7* expression prior and after 2 weeks of anti-TNF treatment were associated with a lack of response in children diagnosed with IBD. Moreover, *TLR2* and *DEFA5* were also differentially expressed in responder patients to anti-TNF drugs when compared to non-responders, but these trends need to be validated in larger cohorts to achieve significant results.

In conclusion, to the best of our knowledge, this is the first study to assess levels of *SMAD7* mRNA in blood as pharmacogenomic markers of early response to anti-TNF drugs in pIBD.

## 4. Materials and Methods

### 4.1. Patient Samples

Between March 2017 and March 2019, 43 IBD patients aged under 18 years were prospectively recruited from the Pediatric Gastroenterology Units of 14 Spanish hospitals. The groups were matched for age and gender. The inclusion criteria were as follows: (1) diagnosis of IBD established based on clinical manifestations, radiological findings, and endoscopic and histological criteria; (2) age 1 to 18 years old; and (3) start of treatment with infliximab (induction regimen of 3 doses [0, 2 and 6 weeks] at 5 mg/kg) or adalimumab (<40 kg: induction with first dose of 80 mg at week 0 followed by a second dose of 40 mg at week 2 and maintenance treatment of 40 mg every two weeks. In patients weighing <40 kg these doses were decreased by half). Demographic and clinical information were also collected. Disease activity was assessed using the Pediatric Crohn’s Disease Activity Index (PCDAI) and Pediatric Ulcerative Colitis Activity Index (PUCAI). Patients were classified as responders and non-responders to anti-TNF therapy based on changes in the PCDAI or PUCAI. Response was defined as a decrease higher than 15 points in any of these indexes from week 0 to 10 for infliximab or from week 0 to 26 for adalimumab. Response was defined as reaching a value of 0 in patients with a value lower than 15 points prior to treatment in any of these indexes.

### 4.2. Ethics Statement

The present study was approved by the Ethic Committee of the Hospital General Universitario Gregorio Marañón and the other participating hospitals (approval number: LLF-TNF-2016-01, 20 January 2017). All patients from 6 to 18 years old assented and parents or legal representatives provided their written informed consent to participate, in accordance with relevant guidelines and regulations.

### 4.3. Extraction of Total RNA from Whole Blood

A blood sample was collected in PAXgene Blood RNA tubes (PreAnalytiX, Hombrechtikon, Switzerland) before the administration of the anti-TNF agent (week 0) and after 2 weeks of treatment (week 2). Total whole blood RNA was extracted using PAXgene Blood RNA kits (PreAnalitiX) according to the manufacturer’s instructions. RNA concentrations were measured using the Quawell Q5000 Spectrophotometer (Quawell Technology Inc, San Jose, CA, USA). RNA integrity was verified using the Agilent RNA 6000 Nano Kit in a 2100 Bioanalyzer (Agilent Technologies, Santa Clara, CA, USA). Extracted RNA was stored at −80 °C until further processing. All RNA samples fulfilled both of the following criteria: RNA integrity ˃ 7 and an optical density ratio of absorbance at 260/280 of between 1.8 and 2.

### 4.4. Quantitative Reverse Transcription-Polymerase Chain Reaction (qRT-PCR)

Total RNA was reverse transcribed using a High Capacity cDNA Reverse Transcription kit (Applied Biosystems, Foster City, CA, USA) following the manufacturer’s instructions. A 1:20 dilution of each cDNA synthesized from 1 µg of total RNA in a volume of 20 µL was used. Expression of the selected genes was quantitated using qPCRBIO SyGreen™ Mix (PCR Biosystems; London, UK) and a StepOnePlus real-time PCR system (Applied Biosystems). Gene expression was calculated using the 2^−∆∆^Ct method. *GAPDH* and *HPRT1* were used for normalization. Real-time PCR was performed in triplicate using 2 μL/well of the cDNA dilution, and 0.04 μM *SMAD7*, *TNF*, *TLR2*, *TNFRSF1B*, *TBX21*, *DEFA5*, *IL11*, *GAPDH*, and *HPRT1* (see Table 2 for primer sequences). In total, the cycling parameters for PCR were 10 min for 95 °C, followed by 40 cycles of denaturation at 95 °C for 15 s and annealing and extension at 60 °C for 60 s. The specificity of the DNA amplifications was verified by melting curve analysis.

### 4.5. Statistical Analysis

Continuous clinical and demographic variables were expressed as the mean and standard deviation (SD) or as the median and interquartile range (IQR); qualitative variables were presented as absolute and relative frequencies. The Fisher exact test or *t*-test was used to compare qualitative and quantitative variables, respectively. 

Gene expression was quantified using ExpressionSuite v1.1 (Applied Biosystems, Foster City, CA, USA) and data were reported as the mean relative quantification and maximum and minimum relative quantification; all the assays were run in triplicate. The unpaired *t*-test was applied to compare gene expression between groups, with a confidence level of 95% and maximum Ct of 35. Efficiency for each primer pair probe was calculated and used for correction. A *p* value < 0.05 was considered statistically significant. The statistical analyses were performed using ExpressionSuite v1.1 (Applied Biosystems, Foster City, CA, USA) and GraphPad Prism v5.1 (GraphPad Software, San Diego, CA, United States).

The PPV, NPV, sensitivity, specificity and diagnostic odds ratios for *SMAD7* relative expression were calculated as previously described [51]. Likelihood ratio + and—were calculated with a CI of 95%.

## Figures and Tables

**Table 1:** Characteristics of patients.

Characteristic	Overall (*n* = 33)	Responders (*n* = 27)	Non-Responders (*n* = 6)	*p* Value
**Gender**				
Male, *n* (%)	16 (48.5%)	12 (44.4%)	4 (66.7%)	0.398
Female, *n* (%)	17 (51.5%)	15 (55.6%)	2 (33.3%)	
**Age (years)**				
At diagnosis, median (IQR, range)	10.4 (4.6; 0.7–17)	10.5 (4.7; 6.5–17)	10.1 (7.2; 0.7–13.0)	0.276
At start of treatment, median (IQR, range)	12 (4; 1.1–17)	12(4.8; 7.9–17)	11.4 (6.2; 1.1–14.1)	0.342
**Type of IBD**				
CD, *n* (%)	26 (78.8%)	21 (77.8%)	5 (83.3%)	1
UC, *n* (%)	7 (21.1%)	6 (22.2%)	1 (16.2%)	
**Type of Anti-TNF**				
Infliximab, *n* (%)	18 (54.5%)	14 (51.9%)	4 (66.7%)	0.665
Adalimumab, *n* (%)	15 (45.5%)	13 (48.1%)	2 (33.3%)	
**PCDAI at start of treatment, median (IQR, range)**	28.8 (25.6; 5–60)	30 (28.8; 5–60)	15 (12.5; 7.5–30)	0.013 **
**PUCAI at start of treatment, median (IQR, range)**	45 (40; 5–60)	50 (43.8; 5–60)	45 *	-
**C-reactive protein at start of treatment, median (IQR, range)**	22 (31.6; 0–150.3)	22.6 (41.3; 0–105.3)	10.6 (19.1; 6.1–27.5)	0.054
**Other treatments**				
Enteral nutrition	14 (42.4%)	12 (44.4%)	2 (33.3%)	0.682
Corticosteroids	12 (36.4%)	10 (37%)	2 (33.3%)	1
Azathioprine	20 (60.6%)	16 (59.3%)	4 (66.7%)	1
Aminosalicylates	12 (36.4%)	11 (40.7%)	2 (33.3%)	0.379
Methotrexate	3 (9.1%)	2 (7.4%)	1 (16.7%)	1
Tacrolimus	2 (6.1%)	1 (3.7%)	1 (16.7%)	0.335
Adalimumab	1 (3%)	0	1 (16.7%)	0.182
Infliximab	1 (3%)	0	1 (16.7%)	0.182

IBD, inflammatory bowel disease; CD, Crohn’s disease; UC, Ulcerative Colitis; IQR, interquartile range; PCDAI. Pediatric Crohn’s Disease Activity Index; PUCAI. and Pediatric Ulcerative Colitis Activity Index; * IQR Not applicable. ** *p* value < 0.05.

**Table 2:** Oligonucleotide sequences used for PCR amplification.

Gene	Forward (5′–3′)	Reverse (5′–3′)
*SMAD7*	ACCCGATGGATTTTCTCAA	AGGGGCCAGATAATTCGTTC
*TNF*	AGCCCATGTTGTAGCAAACC	TCTCAGTCTCACGCCATT
*TLR2*	TGTCATTCTTTCTTCCTGCTAAGA	CTAGGTAGGACAGAGAATGCCTTT
*TNFRSF1B*	AGGCCACCATATTCAGTGCT	GCAGATTTCTAGTTAGAAGTGCGTTA
*TBX21*	CCCAACTGTCAATTCCTTGG	GGGAACAGGATACTGGTTGG
*DEFA5*	TCAGCTCTTTCCTGGAGTGAC	AGGACCATCGCCATCCTT
*IL11*	GGACAGGGAAGGGTTAAAGG	GCTCAGCACGACCAGGAC
*TREM1*	GATGCTCTTTGTCTCAGAAC	CTCTCCGTCCCTTATTATCTG
*OSM*	GTACTGCTCACACAGAGG	TATATAGGGGTCCAGGAGTC
*GAPDH*	AGCCACATCGCTCAGACAC	GCCCAATACGACCAAATCC
*HPRT1*	GACCAGTCAACAGGGGACAT	GTGTCAATTATATCTTCCACAATCAAG

## Supplementary Materials

The following are available online at https://www.mdpi.com/1422-0067/21/9/3364/s1, Table S1: Comparison of relative expression of the TLR2, TNF, TNFRSF1B, IL11, TBX21, SMAD7 and DEFA5 genes in responders vs. non-responders before initiation of anti-TNF treatment., Table S2: Relative expression of the TLR2, TNF, TNFRSF1B, IL11, TBX21, SMAD7, and DEFA5 genes in responders vs. non-responders at week 2.

## Abbreviations

IBD	Inflammatory bowel disease
pIBD	Pediatric inflammatory bowel disease
UC	Ulcerative colitis
CD	Crohn’s disease
SD	Standard deviation
IQR	Interquartile range
PCDAI	PCDAI. Pediatric Crohn’s Disease Activity Index
PUCAI	Pediatric Ulcerative Colitis Activity Index
SEM	Standard error of the mean
PPV	Positive predictive value
SEM	Standard error of the mean
PPV	Positive predictive value
NPV	Negative predictive value
