# Supplementary material for: Gene Signatures of Early Response to Anti-TNF Drugs in Pediatric Inflammatory Bowel Disease
Source: Int J Mol Sci. 2020 May 9;21(9):3364. doi: 10.3390/ijms21093364 (PMC7247673; doi:10.3390/ijms21093364)
Supplement: Supplementary file 1 [file ijms-21-03364-s001.pdf]

Supplemental Table S1. Comparison of relative expression of the *TLR2, TNF, TNFRSF1B, IL11, TBX21, SMAD7* and *DEFA5* genes in responders vs. non-responders before initiation of anti-TNF treatment.

Gene symbol	Gene name	RQ (RQmin-RQmax)	*P* value
TLR2	Toll-like receptor 2	0.542 (0.294-0.999)	0.056
TNF	Tumor necrosis factor	1.028 (0.78-1.354)	0.851
TNFRSF1B	TNF receptor superfamily, member 1B	0.879 (0.689-1.123)	0.325
IL11	Interleukin 11	1.448 (0.48-4.37)	0.441
TBX21	T-cell-specific T-box	0.882 (0.34-2.286)	0.769
SMAD7	SMAD family member 7	0.486 (0.248-0.953)	**0.040**
DEFA5	Defensin A5	0.499 (0.173-1.441)	0.177
TREM1	Triggering Receptor Expressed On Myeloid Cells 1	1.051 (0.842-1.312)	0.728
OSM	Oncostatin M	0.965 (0.624-1.493)	0.854

RQ, relative quantification; Responders were used as reference for comparison

Supplemental Table S2. Relative expression of the *TLR2, TNF, TNFRSF1B, IL11, TBX21, SMAD7,* and *DEFA5* genes in responders vs. non-responders at week 2.

Gene symbol	Gene name	RQ (RQmin-RQmax)	*P* value
TLR2	Toll-like receptor 2	0.614 (0.374-1.008)	0.082
TNF	Tumor necrosis factor	1.149 (0.925-1.427)	0.216
TNFRSF1B	TNF receptor superfamily, member 1B	0.977 (0.738-1.293)	0.859
IL11	Interleukin 11	1.623 (0.722-3.652)	0.204
TBX21	T-cell-specific T-box	1.156 (0.604-2.214)	0.637
SMAD7	SMAD family member 7	0.449 (0.25-0.995)	**<0.049**
DEFA5	Defensin A5	0.881 (0.392-1.98)	0.756
TREM1	Triggering Receptor Expressed On Myeloid Cells 1	0.982 (0.624-1.547)	0.931
OSM	Oncostatin M	0.898 (0.534-1.513)	0.63

[1] RQ, relative quantification. Responders were used as reference for comparison
